# Retraction: Tramadol-induced hepato- and nephrotoxicity in rats: Role of Curcumin and Gallic acid as antioxidants

**DOI:** 10.1371/journal.pone.0347088

**Published:** 2026-04-13

**Authors:** The PLOS One Editors

Following the publication of this article [1], concerns were raised about the results presented in Figs 1 and 2. Specifically,

- The following panels appear to partially or fully overlap, despite being used to represent different experimental conditions or samples obtained from different animal species, calling into question the reliability of the associated quantified results:
- All β-actin panels in Fig 1 of this article [1], all β-actin panels in Figs 1 of [2] and [3, retracted in 4], and all β-actin panels in Figs 2 and 3 of [5, retracted in 6].
- Fig 2C and Fig 2I.

The authors did not respond to editorial requests for comment or to provide the underlying data.

In light of these concerns, which call into question the integrity and reliability of the published results, the *PLOS One* Editors retract this article.

SAS did not agree with the retraction. AAA and SGEB either did not respond directly or could not be reached.
